# Double Layer Composite Electrode Strategy for Efficient Perovskite Solar Cells with Excellent Reverse-Bias Stability

**DOI:** 10.1007/s40820-022-00985-4

**Published:** 2022-12-13

**Authors:** Chaofan Jiang, Junjie Zhou, Hang Li, Liguo Tan, Minghao Li, Wolfgang Tress, Liming Ding, Michael Grätzel, Chenyi Yi

**Affiliations:** 1 State Key Laboratory of Power System, Department of Electrical Engineering, Tsinghua University, Beijing, 100084 People’s Republic of China; 2 Institute of Computational Physics (ICP), ZHAW School of Engineering, Wildbachstr. 21, 8400 Winterthur, Switzerland; 3 Center for Excellence in Nanoscience (CAS), Key Laboratory of Nanosystem and Hierarchical Fabrication (CAS), National Center for Nanoscience and Technology, Beijing, 100190 People’s Republic of China; 4 Laboratory of Photonics and Interfaces, Department of Chemistry and Chemical Engineering, Swiss Federal Institute of Technology Lausanne, 1015 Lausanne, Switzerland

**Keywords:** Composite electrode, Perovskite solar cells, Stability, Reverse bias, Characterization

## Abstract

Perovskite solar cells (PSCs) have become the representatives of next generation of photovoltaics; nevertheless, their stability is insufficient for large scale deployment, particularly the reverse bias stability. Here, we propose a transparent conducting oxide (TCO) and low-cost metal composite electrode to improve the stability of PSCs without sacrificing the efficiency. The TCO can block ion migrations and chemical reactions between the metal and perovskite, while the metal greatly enhances the conductivity of the composite electrode. As a result, composite electrode-PSCs achieved a power conversion efficiency (PCE) of 23.7% (certified 23.2%) and exhibited excellent stability, maintaining 95% of the initial PCE when applying a reverse bias of 4.0 V for 60 s and over 92% of the initial PCE after 1000 h continuous light soaking. This composite electrode strategy can be extended to different combinations of TCOs and metals. It opens a new avenue for improving the stability of PSCs.

**Supplementary Information:**

The online version contains supplementary material available at 10.1007/s40820-022-00985-4.

## Highlights

- A composite electrode strategy to fabricate the perovskite solar cells (PSCs) with excellent comprehensive stabilities, particularly reverse-bias stability.
- A record efficiency of 23.7% (certified 23.2%) for* n-i-p* PSCs using Cu as the electrode.
- The strategy can be extended to the combinations of different transparent conducting oxides and low-cost metals.

**Supplementary Information:**

The online version contains supplementary material available at 10.1007/s40820-022-00985-4.

## Graphical abstract

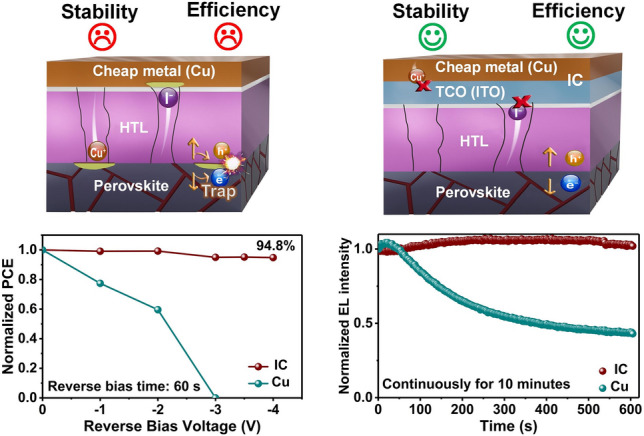

## Introduction

Perovskite materials have achieved good performance in optoelectronic devices such as perovskite solar cells (PSCs) and light emitting diodes (LEDs) [1–4]. However, the stability of perovskite optoelectronic devices is still not satisfactory for large-scale applications [5, 6], especially in terms of stability in reverse bias [7]. Solar cells can become partially shaded under various circumstances, such as the shadow of cloud, nearby trees and bird droppings. The shaded cells in a solar module end up in reverse bias by being forced to pass the photocurrent of its unshaded neighbors. The unfavorable reverse current flow can cause highly conductive pathways between electrodes (shunts), resulting in excessive local heating which may cause damage to the cells and encapsulants [7, 8]. A protocol has been set by International Electrotechnical Commission (IEC) standards (IEC 61646 and IEC 61215) to test the stability of photovoltaic (PV) modules under reverse bias for evaluating the partial shading resilience of PV modules [9]. Silicon cells usually breakdown in reverse bias because of avalanche breakdown and the breakdown voltage (*V*_BD_) is typically more than 15 V. In comparison, PSCs exhibit higher density of mobile ions and stronger capacitance effect. The accumulated mobile ions in PSCs under illumination with high reverse bias voltage is easier to tunnel through the perovskite layer to form localized shunts, leading to much lower *V*_BD_ [10]. Because of the high efficiency and low cost, PSCs hold great promise for PV markets, nevertheless, unsatisfactory reverse bias stability severely restricts it from large scale application. Thus, it is urgent to achieve adequate stability under reverse bias.

McGehee et al. [10] studied the relationship between ion migration and reverse-bias stability. They demonstrated that the breakdown current in reverse bias is most likely to originate from the tunneling mediated by mobile ions, which indicates that the reverse-bias stability has great correlations with interlayer ion migrations between the perovskite and electrodes [11]. Irreversible chemical reactions between the metal electrode and perovskite layer can appear and can deteriorate perovskite film and adjacent layers. For instance, metal ions from the electrode can diffuse to the perovskite layer under reverse bias, resulting in deep-level defects in the perovskite layer and undesirable S-shape curves in performance test [12–15]. For the PSCs under light irradiation and the electric field, iodine ions can diffuse toward the metal electrode, leaving iodine vacancy defects in the perovskite [16, 17]. Moreover, migrated iodine can react with the metal electrode to generate metal iodide, which increases the series resistance of the PSCs, resulting in PCE decay [18, 19]. Thus, it is urgent to find an effective way to prevent interlayer ion migration and chemical reactions between metals and perovskites without sacrificing the PCE [20, 21].

**Fig. 1:**
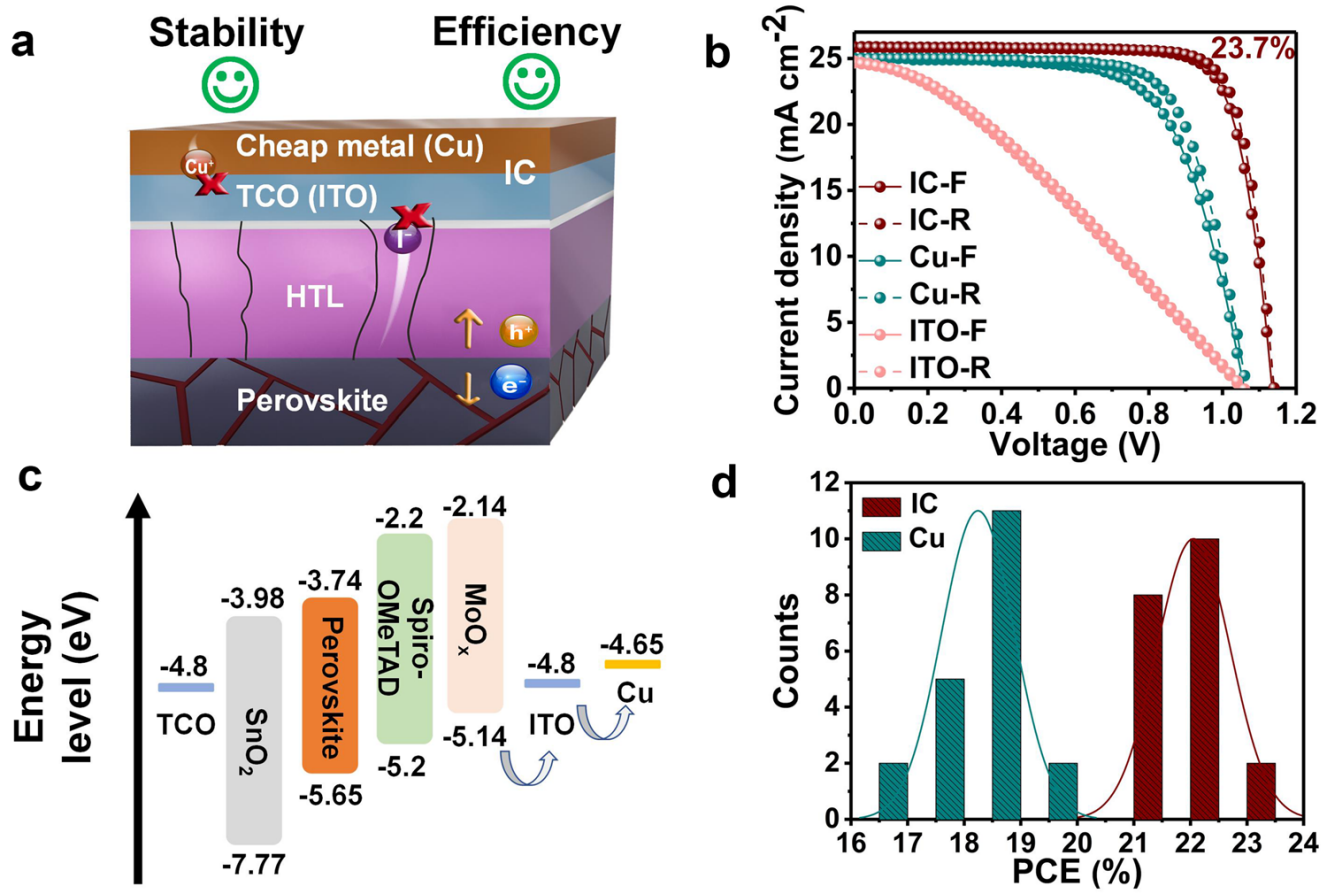
Schematic illustration of different electrodes and performances of corresponding devices. **a** Schematic illustration of TCO in addition to charge transport in composite electrode (i) block metal ion diffusion; (ii) prevent ion diffusion from perovskite and related chemical reactions. **b** Current density–voltage curves for the best performance PSCs with different electrodes. **c** Diagram of the energy levels of the different layers in PSCs. **d** Histograms of the PCEs for reference (Cu) PSCs and IC-PSCs

Herein, we proposed a composite electrode strategy to fabricate efficient PSCs with excellent reverse-bias stability (Fig. 1a). We substituted the commonly-used gold electrode with the copper (Cu) because of its low price and uncompromised conductivity. The price of Cu (~ 0.01 USD g^−1^) is two and four orders of magnitude lower than of Ag (~ 0.64 USD g^−1^) and Au (~ 53.29 USD g^−1^), respectively [22–24]. The TCO can effectively block ion migrations and chemical reactions between the metal and perovskite, while Cu can greatly enhance the conductivities of the composite electrode [25, 26]. The combination of TCO with low-cost metal (i.e., ITO + Cu, named as IC) leads to composite electrodes with excellent conductivity and stability (Fig. 1a). We applied the IC composite electrode to PSCs and achieved a champion PCE of 23.7% (certified PCE 23.2%), which is the record efficiency for* n-i-p* PSCs with low cost metal Cu as the electrode. Moreover, the PSCs exhibited excellent stabilities under different stressing conditions. The PSCs with composite electrodes demonstrate a stable electroluminescence output under a forward bias of 1.3 V and can maintain 95% of the initial PCE after holding at a reverse bias of 4.0 V for 60 s. In addition, the unencapsulated PSCs maintained 92.1% of its initial PCE after 1000 h of continuous light soaking in N_2_ under MPPT and maintained 96.6% of its initial efficiency after continuous heating for 500 h. Furthermore, this strategy can be extended to the combinations of different TCOs (i.e., ITO, IZO, AZO) and low-cost metals (i.e., Cu, Al, Ni). Systematic investigation of the aged devices reveals the mechanism of the better stability of the composite electrode. The composite electrode not only prevents interlayer ion diffusion but also enhances the PCEs of PSCs. Our composite electrode strategy opens a new venue for high-efficiency stable perovskite optoelectronics with low-cost in the future.

## Experimental and Calculation

### Device Fabrication

The cleaned ITO glass substrates were treated with the UV-ozone before spin-coating SnO_2_ solution (4000 rpm for 30 s). Afterwards, the substrates were annealed at 150 °C for 30 min in ambient air. Another UV-ozone was done before depositing perovskite films. The perovskite films were fabricated by typical two-step method according to our former report [27]. Subsequently, a thin layer of hole transporting film was deposited by spin coating spiro-OMeTAD solution. Then, 10 nm MoO_*x*_ was thermally evaporated as the buffer layer. For the composite electrode, indium tin oxide (ITO) was deposited onto MoO_*x*_ by magnetron radio frequency (RF) sputtering with a cylindrical ITO target. Finally, 100 nm of low-cost metal (Cu/Al/Ni) was thermally evaporated onto TCO to form the composite electrode.

### Materials Characterization

The SEM images and EDS mapping were taken with a Hitachi SU8010 instrument. The XRD patterns were measured by the device of Rigaku SmartLab (copper Kα, λ: 1.54 Å, 150 mA, 40 kV). XPS spectra were measured by an ESCALAB 250Xi system (Thermo Fisher Scientific) with aluminium Kα X-ray radiation. AFM images were obtained with a Dimension Icon, Bruker system. Auger electronic spectrum (AES) was tested with a model PHI-700 nano scanning auger system (ULVAC-PHI company, Japan). EL, TPV, TPC and EQE were obtained from the Cicci test platform, Italy.

### Device Measurement

The active area is 0.24 cm^2^ and the aperture area is 0.1 cm^2^. Photocurrent voltage (*J–V*) curves were obtained from a solar simulator (Newport, Oriel Class AAA, 94,043 A) and matched with the Keithley 2400 source meter. The light intensity was calibrated with standard silicon reference certified by NREL. The voltage scan rate was 50 mV s^−1^ with a step voltage of 20 mV. The operational stability was tested with a homemade MPP tracking instrument under continuous illumination with a white LED lamp source, at 25 °C, in N_2_ condition. The reverse-bias stability of the devices was recorded by holding the device for a designated time at the respective reverse bias voltage in the dark before *J–V* sweeping under AM 1.5 G illumination.

## Results and Discussion

### Device Performances of the PSCs

Indium tin oxide (ITO) and Cu were selected as representative for the composite electrode. The device structure is ITO/SnO_2_/perovskite/spiro-OMeTAD/MoO_*x*_/IC (Fig. S1a), MoO_*x*_ is used to protect the perovskite film during ITO sputtering [28]. For comparison, reference PSCs with only Cu as the counter electrode were fabricated (Fig. S1b). The cross-sectional SEM images clearly showed that with an additional ITO layer, the Cu layer of the composite IC electrode was smoother than that of the bare Cu electrode. The smoother Cu film can reflect light more efficiently, which can be helpful to harvest long wavelength light that is not completely absorbed before reflection.

The* n-i-p* PSCs were fabricated by a conventional two-step method [29] with IC and Cu as counter electrodes for PSCs with IC composite electrodes (IC-PSCs) and reference PSCs, respectively. We achieved a champion PCE of 23.7% (certified PCE of 23.2% in Fig. S2) and 19.0%, with an open-circuit voltage (*V*_oc_) of 1.14 and 1.07 V, short-circuit current density (*J*_sc_) of 25.84 and 25.01 mA cm^−2^, and fill factor (FF) of 80.29 and 71.25% under optimized conditions for IC-PSCs and reference PSCs, respectively, as shown in Fig. 1b and Table 1. Compared with the reference PSCs, the *J*_sc_ of IC-PSCs increased by 0.83 mA cm^−2^, the *V*_oc_ increased by 70 mV, and the FF increased by 12.69%. We attribute the higher *J*_sc_ of IC-PSCs to better light utilization, the higher *V*_oc_ to a better energy level alignment [30] (Fig. 1c) and reduced nonradiative charge recombinations with the insertion of ITO between Spiro-OMeTAD and Cu [31, 32], and the higher FF to the larger shunt resistance, as indicated by the lower leakage current (Figs. S3 and S4). Comparison of the forward and reverse scanning results shows that the hysteresis index of IC-PSC is much smaller than that of the reference PSC (0.02 vs 0.07), which is probably due to the better charge transport in the IC-PSCs [33]. To further compare the reproducibility of different PSCs, we fabricated a batch of 20 devices each with IC and bare Cu electrodes. As shown in Figs. 1d and S5, the reproducibility and overall performance of IC-PSCs (average PCE: 22.05%) are much better than those of the reference PSCs (average PCE: 18.24%). To verify the effect of ITO sputtering, we fabricated PSCs with bare ITO as the electrode. For PSCs with a bare ITO electrode (ITO-PSC), although the FF of the device is only 32.0% due to the large lateral resistance of ITO, which contributes to the series resistance of the PSC [34], its *V*_oc_ and *J*_sc_ are close to that of the reference PSC. This means that the ITO sputtering process does not damage the underlying perovskite film and has no negative effect on the key performance parameters of the PSCs. The optimization of ITO thickness is shown in Table S1 and Fig. S6.

**Table 1:** The best performances of PSCs with different electrodes

Device	*J*_sc_ (mA cm^−2^)	*V*_oc_ (V)	FF (%)	PCE (%)	Hysteresis	*R*_s_ (Ω cm^−2^)
TCO/SnO_2_/Perovskite/SpiroOMeTAD/MoO_*x*_/ITO						
Forward	22.71	1.08	34.15	8.4	0.02	28.7
Reverse	22.62	1.08	33.79	8.2		
TCO/SnO_2_/Perovskite/SpiroOMeTAD/MoO_*x*_/Cu						
Forward	23.73	1.09	73.68	19.1	0.09	2.9
Reverse	23.72	1.08	68.53	17.6		
TCO/SnO_2_/Perovskite/SpiroOMeTAD/MoO_*x*_/ITO + Cu						
Forward	25.88	1.13	79.00	23.2	0.02	2.2
Reverse	25.84	1.14	80.29	23.7		

### Characterizations of the Carrier Dynamics of the PSCs

**Fig. 2:**
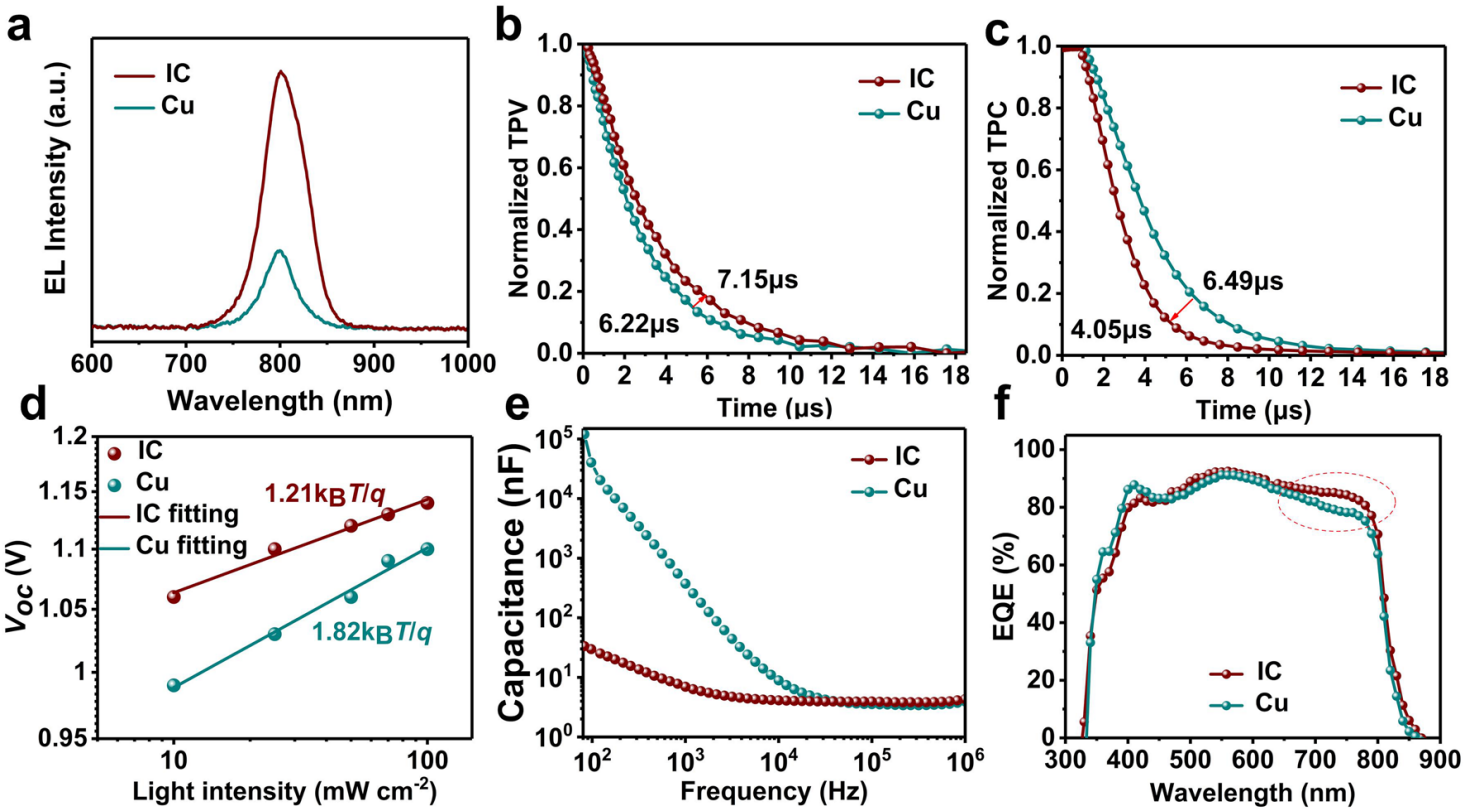
Device characterization and carrier dynamic analysis. **a** EL spectral response of the IC-PSC and reference PSC. Transient photovoltage (**b**) and transient photocurrent (**c**) of the IC-PSC and reference PSC. **d** *V*_oc_ upon light intensity-modulated *J–V* measurement of the IC-PSC and reference PSC. **e** Capacitance-frequency (C-F) test of the complete device under darkness and 0 V bias steady-state conditions. **f** EQE spectral response of the IC-PSC and reference PSC

The impacts of composite electrode on charge carrier recombination were investigated by optoelectrical and electrical characterizations, including electroluminescence (EL), capacitance frequency (C-F) test, transient photovoltage (TPC) and transient photocurrent (TPV) decay under short and open circuit conditions. As depicted in Fig. 2a, the emission peaks of the PSCs are located at 800 nm, and the peak intensity of IC-PSC is approximately 3.4 times higher than that of the reference indicating a reduced nonradiative recombination close to the perovskite-HTL interface, which is consistent with the higher *V*_oc_ for the IC-PSC. TPV and TPC measurements were applied to explore the carrier dynamics of the devices. As shown in Fig. 2b, c, the carrier lifetime of IC-PSC is longer than that of the reference PSC (7.15 vs 6.22 μs) indicating less carrier traps and trap-mediated charge recombination occurred in IC-PSC than in the reference PSC, which is in accordance with the enhanced EL intensity (Fig. 2a). Meanwhile, the photocurrent decay of IC-PSC is faster than that of the reference PSC (4.05 vs 6.49 μs) which indicates better carrier extraction of the IC electrode than the Cu electrode. These can be attributed to the suppression of ion migrations and a more efficient charge extraction by ITO layer in IC-PSCs. Then, we tested the open circuit voltage of the device at different light intensities, as shown in Fig. 2d, the ideality factors of the IC-PSC and reference PSC are 1.21 and 1.82, respectively, indicating reduced trap-assisted SRH recombination in IC-PSC compared with the reference PSC. The frequency response in capacity of the IC-PSC is three orders of magnitude lower than that of the reference in C-F test (Fig. 2e), which demonstrates that the composite electrode can effectively reduce ion migrations and interfacial charge accumulations [18]. The external quantum efficiency (EQE) of the PSCs was characterized to reveal the photon to electron conversion efficiency at different wavelength. The EQE of the IC-PSC is higher than that of the reference PSC in the wavelength range from 650 to 800 nm, as shown in Fig. 2f. It might be due to the better light reflection property of the ITO or Cu of the IC electrode because of smoother interface, which is beneficial to harvest the light that is not completely absorbed by perovskite layer. This is in good agreement with the higher *J*_sc_ of IC-PSCs.

### Device Stability Performance and Analysis

**Fig. 3:**
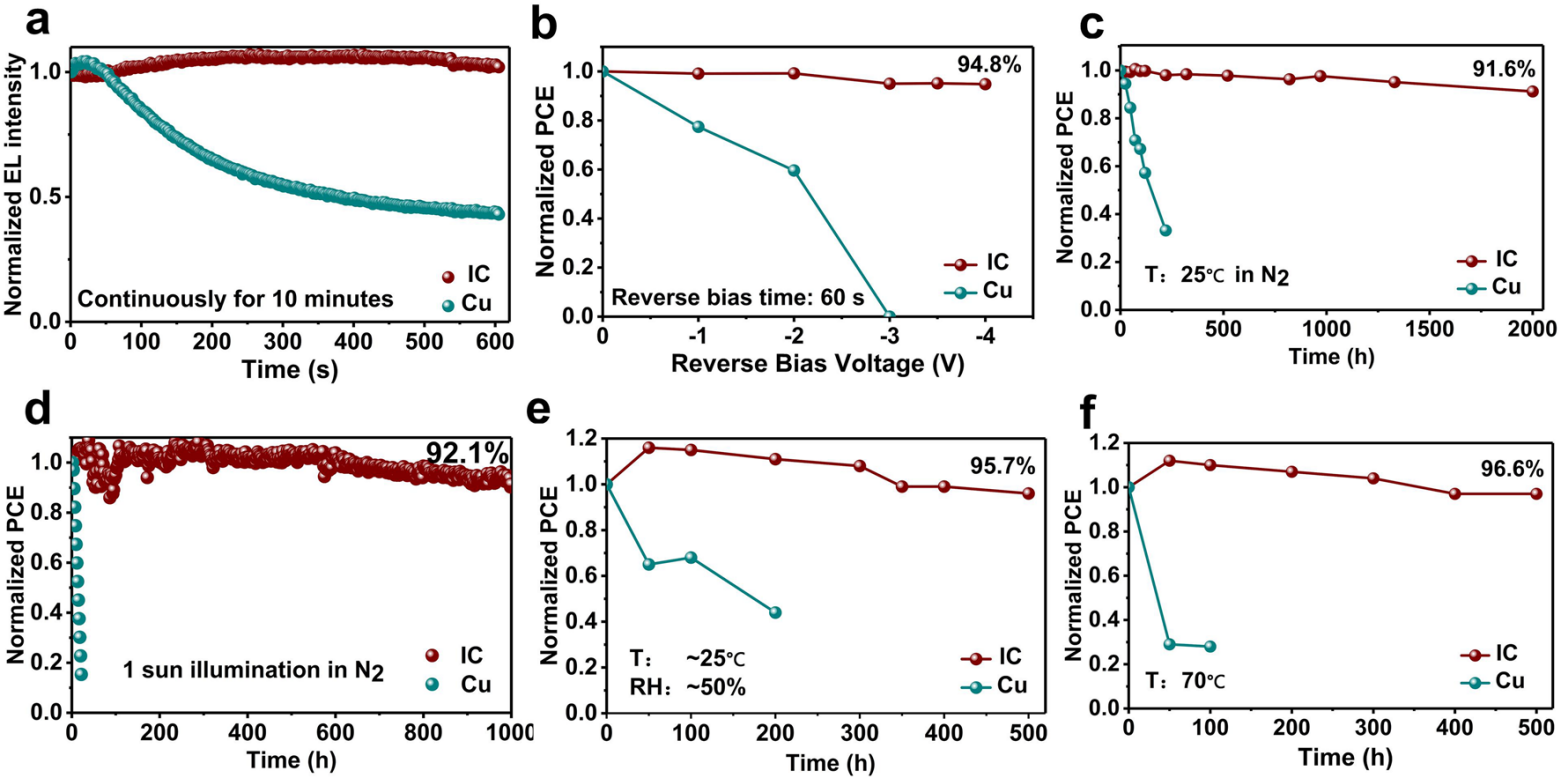
Comprehensive stability performance of unencapsulated PSCs. **a** EL stability performance of IC-PSCs and reference PSCs. **b** Reverse-bias stability performance of IC-PSC and reference PSC tested by *J–V* sweeping after 60 s under different reverse biases. **c** The long-term stability performance of the IC-PSC and reference PSC in N_2_ (25 °C). **d** The MPP tracking performance of the IC-PSC and reference PSC under continuous light illumination (white LED, neither cooling nor UV filters were used in this test). **e** Humidity stability performance of IC-PSC and reference PSC (relative humidity, ~ 50%). **f** Thermal stability performance of IC-PSC and reference PSC aged at 70 °C in a glove box

The IC-PSCs demonstrated all-round stability enhancement compared with the reference PSCs. We tested the stability of the PSCs in an electric field by applying forward bias (electroluminescence, EL) and reverse bias voltages on the PSCs. The EL peak intensity of IC-PSC hardly reduced and maintained a very strong electroluminescence after continuous operation for 600 s under a forward bias of 1.3 V. In contrast, an obvious decay was observed for the reference device, which maintained only 40% of the initial intensity under the same conditions (Fig. 3a). The results clearly showed the superior electrical stability of IC-PSCs. The outstanding electrical stability under forward bias indicates that the IC composite electrode can be applied not only on PSCs but also on perovskite LEDs and other optoelectronic devices [9]. To further evaluate the reverse-bias stability of the IC-PSCs, we performed the test by holding the devices at different voltages for a certain time before *J–V* sweeping (Fig. 3b). As shown in Fig. S7, the reference device maintained only 18% of its initial PCE after holding at a reverse bias of 1.0 V for 120 s before *J–V* sweeping. In comparison, the IC-PSC maintained 95% of its initial PCE under the same conditions, demonstrating the superior reverse bias stability of PSCs with composite electrode. We then investigated the evolution of PSC performances under higher reverse bias. The reference devices retained only 60% of its initial PCE and completely lost its PCE after holding at 2.0 and 3.0 V for 60 s. In comparison, the IC-PSC showed negligible PCE decay at 3.0 V reverse bias and even maintained 95% of its initial PCE after holding at 4.0 V for 60 s, demonstrating excellent reverse bias stability for the PSCs with composite electrode [8].

The IC-PSCs also demonstrated good stabilities under other (stress) conditions. We tracked the long-term shelf-life stability of unencapsulated PSCs stored in nitrogen. The IC-PSC maintained 91.6% of the initial value after 2000 h of storage (Figs. 3c and S8). In contrast, the efficiency of the reference PSC decreased to 57% of its initial value only after 220 h (Figs. 3c and S9) under the same conditions. The decrease of the performance of the reference devices is mainly attributed to the reduction of short-circuit current density and fill factor (Fig. S10). The rapid decay of the performance of the reference PSCs can be related to the low formation enthalpy of CuI (Table S2) and the high reactivity of Cu toward iodine [35]. The exceptional stability of IC-PSC clearly demonstrated the function of ITO as a blocking layer to prevent interlayer ion diffusion and reactions.

Figure 3d shows the stability of the PSCs under maximum power point (MPP) tracking under continuous light soaking in the N_2_ atmosphere. The IC-PSCs maintained more than 92.1% of the initial efficiency after 1000 h of MPP tracking, which is the best operational stability among all “*n-i-p*” PSCs with Cu electrode. In contrast, the reference PSCs decreased to less than 20% of its initial value after only one hour, demonstrating poor operational stability, which can be attributed to the undesirable ion migrations under light soaking, which accelerated the reactions between the perovskite and the Cu. In contrast, the interlayer ion diffusion and subsequent chemical reactions can be effectively inhibited by the ITO barrier in the IC-PSCs, resulting in the outstanding operational stability under continuous light illumination.

We then investigated the stability of the IC-PSCs in humidity and under thermal treatment. The humidity stability of unencapsulated PSCs was tested at room temperature at a relative humidity (RH) of ~ 50%. The IC-PSCs maintained 95.7% of the initial efficiency after 500 h (Fig. 3e), demonstrating enhanced suppression against moisture infiltration of the TCO layer. In comparison, the PCE of the reference PSCs decreased to 40% of the initial value after only 200 h. Heat is another promoter of ion diffusion. We kept the unencapsulated PSCs at 70 °C (under argon atmosphere). The IC-PSCs maintained 96.6% of their initial efficiency after continuous heating for 500 h. In comparison, the reference devices can only maintain less than 30% of their initial value after 50 h (Fig. 3f).

### Elucidating the Underlying Reasons for the Better Stabilities of IC-PSCs

To reveal the underlying reasons for the outstanding performances and stabilities of the IC-PSCs, a series of characterizations were conducted. Thermal image microscopy was performed to evaluate the leakage current in both devices. Obvious hot spots appeared in the reference PSC after 27 cycles under 0.5 V bias, with a leakage current of 1.2 mA (Fig. S3). In contrast, no obvious hot spots were found in IC-PSC (Fig. S4) after 28 cycles under 1.0 V bias, with a leakage current of only 28 nA. The exceptionally low leakage current explains an enhanced electrical stability under bias and larger shunt resistance of IC-PSCs.

**Fig. 4:**
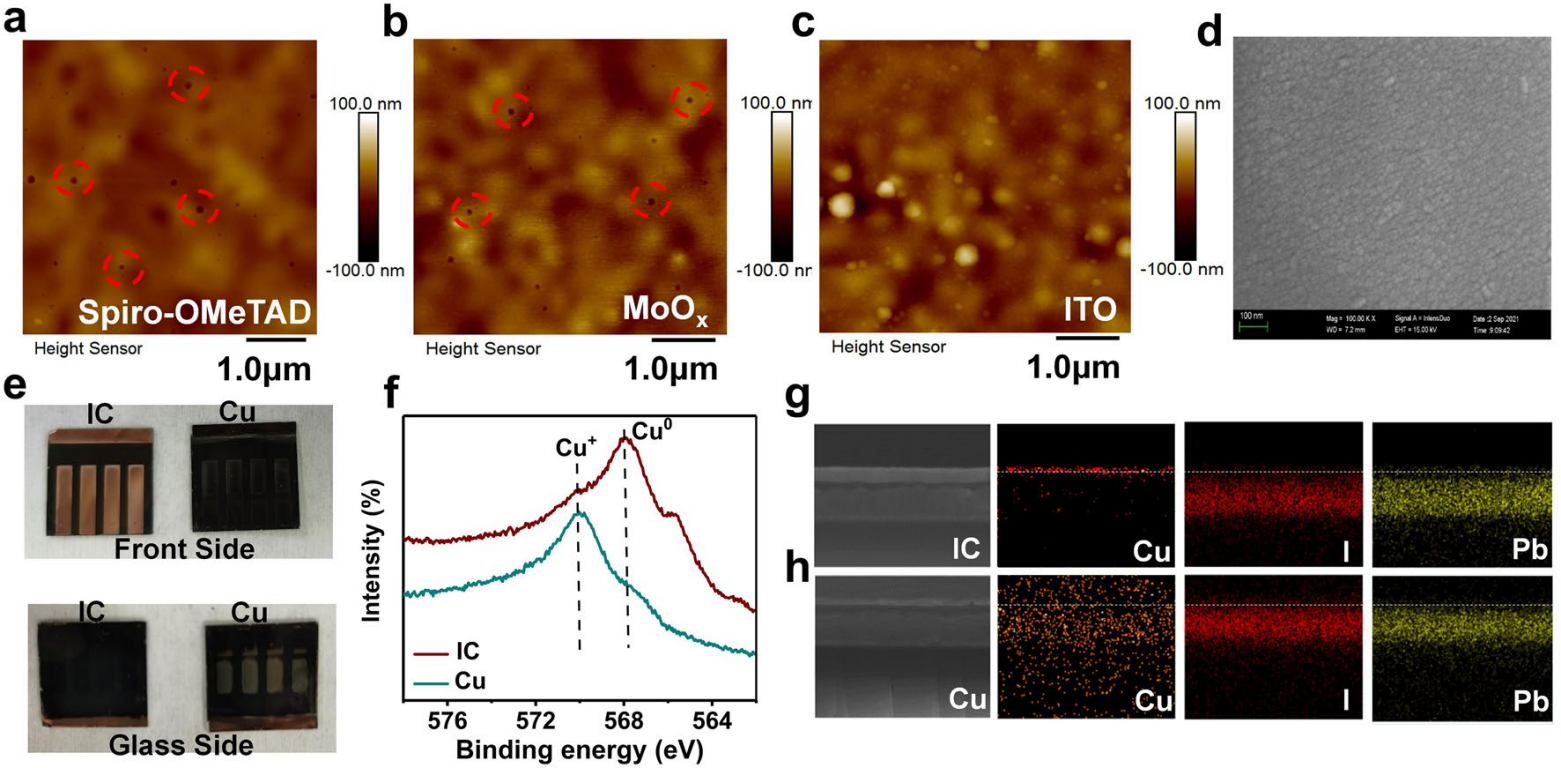
Characterization of the devices. AFM images for the **a** Spiro-OMeTAD, **b** MoO_x_ and **c** ITO surface morphology of the partial PSC device. **d** SEM of the ITO film based on partial PSC. **e** Optical images (front and glass side) of the aged IC-PSC and reference PSC after 1000 h of MPPT. **f**, XPS spectra of the aged PSC electrode surface copper valence state after 1000 h of MPPT. Cross section EDX mapping of Cu, I, and Pb elements for aged IC-PSC (**g**) and reference PSC (**h**) after 1000 h of MPPT

Numerous pinholes were observed in the spiro-OMeTAD and MoO_*x*_ layers for the reference PSC by atomic force microscopy (AFM) test (Figs. 4a, b and S110), while they were completely filled by the ITO layer in the IC-PSC (Fig. 4c), leading to a dense and uniform film as shown by SEM in Fig. 4d.We speculate that the pinholes in spiro-OMeTAD and MoO_*x*_ layers can serve as channels for ion diffusion, which can accommodate the downward diffusion of copper and upward diffusion of iodine, forming defects and resulting in a PCE decrease in the reference devices. The low formation enthalpy (Table S2) of CuI indicates that the copper is prone to reaction with iodine [35], which deteriorates the perovskite films, leading to poor stability and low PCE for the reference devices. In contrast, the compact ITO film can serve as a physical barrier to inhibit undesired ion diffusion and defect generation as well as the subsequent chemical reactions, contributing to the excellent long-term stability of IC-PSCs. The ion diffusion and the resulting degradation can also be evident by the comparison of the optical image of the aged devices (Fig. 4e).

The aged devices (after 1000 h of light soaking) were subjected to a series of in-depth and systematic characterizations to reveal the key role of IC composite electrodes in improving the stability of PSC devices and the underlying degradation mechanism of the reference devices. X-ray photoelectron spectroscopy (XPS) measurements (Fig. 4f) show that after 1000 h of continuous light aging, the IC-PSCs maintained a strong Cu^0^ signal, indicating that the copper in the IC electrode was well protected and did not suffer from chemical reactions. In contrast, for the reference devices, a strong Cu^+^ signal was observed, while the Cu^0^ was seriously diminished. The XPS results show an obvious chemical change from Cu^0^ to Cu^+^ for the copper electrode of the reference PSCs, thus losing its good conductivity.

Cross-sectional energy dispersive X-ray spectroscopy (EDS) was applied to map the distribution of the elements of interest in the aged devices. For the aged IC-PSCs (Fig. 4g), Cu is located on top of the devices. In contrast, Cu is distributed throughout the device in both the electrode and perovskite layers for the aged reference device (Fig. 4h). Moreover, iodine (I) is observed in the electrode layer for the aged reference devices (Fig. 4h). We speculate that the Cu from the electrode diffused through the hole transporting layer into the perovskite layer, and the iodine from the perovskite layer diffused into the electrode layer for the aged reference PSCs. A large amount of Cu can serve as traps in perovskite, and iodine can decrease the conductivity of Cu, which are unfavorable for the photovoltaic performances and long-term stabilities of PSCs.

**Fig. 5:**
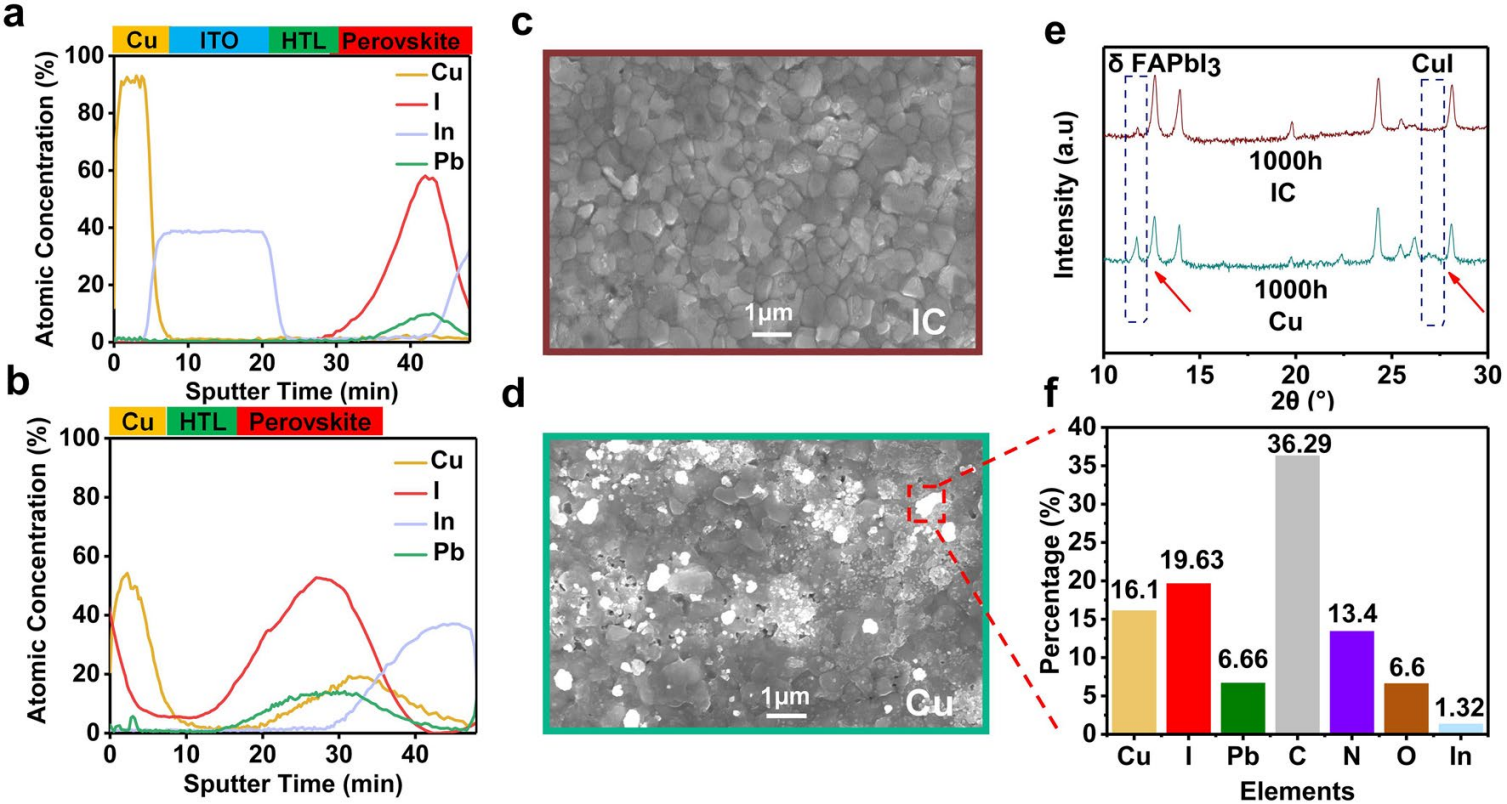
In-depth analysis of aged PSCs after 1000 h of MPPT. AES depth profiles for aged IC-PSC (**a**) and reference PSC (**b**). **c** SEM for the perovskite layer morphology of the aged IC-PSCs. **d** SEM for the perovskite layer morphology of the aged reference PSC. **e** XRD patterns of aged IC-PSC and reference PSC. **f** EDS point mapping element proportions for the white spot on SEM

Auger electron spectroscopy (AES) depth profiling gives an in-depth view of quantitative analysis by tracking the key elements Cu, I, In, and Pb. The AES spectrum can clearly show the functional layers in terms of element distribution and respective concentrations and can visually distinguish the sharp interface of each functional layer, namely perovskite/Spiro-OMeTAD/ITO/Cu. As shown in Figs. 5a and S12, for the IC-PSCs, the copper is localized in the electrode with no obvious signal in other layers after 1000 h of aging under continuous illumination, indicating that the Cu diffusion was effectively suppressed by the ITO barrier in the composite electrode. The AES signals of I and Pb are exclusively detected in the perovskite layer with no obvious diffusion to other layers, showing the ion migrations from perovskite to metal can be blocked by the ITO layer in the composite IC electrode. In contrast, strong diffusion is observed in the aged reference PSCs (Fig. 5b) with obscure interfaces in AES patterns. The Cu concentration is sharply decreased in the electrode layer, while the signal of Cu was considerably increased in the perovskite layer, indicating the diffusion of Cu from the electrode into the perovskite layer. In addition, the iodine concentration significantly increased in the electrode layer, indicating the iodine diffusion from the perovskite layer to the electrode, which is consistent with the XPS and EDS mapping results.

To further understand the degradation mechanism, we carefully removed the electrode with adhesive tape and dissolved the hole transport layer with toluene to expose the perovskite layer of the aged PSCs for further analysis. As shown in Fig. 5c, the morphology of the aged perovskite layer of IC-PSCs looks similar to that of the fresh perovskite films (as shown in Fig. S13), with complete grains and clear boundaries, indicating a well-preserved perovskite film after the aging test. In contrast, the morphology of the aged perovskite layer of the reference PSCs is severely deteriorated (Fig. 5d). Numerous holes were found in the reference perovskite film, and we speculate that the holes are formed due to the loss of *I*^−^ because of the chemical reaction with Cu and the diffusion of I^−^ from perovskite into the electrode layer. In addition, many white spots were observed. We chose one white spot for EDS mapping, which revealed that the proportions of Cu, I and Pb in the spot were 16.10, 19.63, and 6.66%, respectively, as summarized in Fig. 5f. The high concentration of Cu indicates the existence of CuI, which is confirmed by the XRD spectra of the aged reference PSCs (Fig. 5e). In addition, compared with the XRD pattern of the aged IC-PSCs, the reference PSCs shows an intense peak at 11.6°, which can be attributed to the δ-phase FAPbI_3_ (Fig. 5e). The higher amount of δ-phase FAPbI_3_ phase can also contribute to the PCE decay of the aged reference PSCs.

To summarize, the interlayer diffusions of copper from the metal electrode layer and iodine from the perovskite layer occur in reference PSCs, particularly under light irradiation or heating, resulting in the severe degradations of the perovskite and copper electrode. The introduction of ITO in the IC electrode can effectively block the interlayer ion diffusion and the consequent chemical reactions, suppressing the formation of defects under external stress. As a result, the IC-PSCs showed exceptional overall stabilities under various harsh environmental conditions.

### Generality of the Composite Electrode Strategy

We further tested the generality of the composite electrode strategy by using different combinations of TCO materials (i.e., ITO, IZO, AZO) and low-cost metals (i.e., Cu, Al, Ni) for composite electrodes in IC-PSCs. Figure S14 shows the *J–V* curves of PSCs with different composite electrodes and Table S3 summarizes the photovoltaic performances. Based on the combination of Cu with IZO and AZO, two composite electrodes, namely IZO + Cu (IZC) and AZO + Cu (AZC), were produced. The preliminary tests showed that after 400 h of continuous light soaking, devices with IZC and AZC composite electrodes maintained 87.7 and 86.8% of their initial PCE, respectively (Figs. S15 and S16). The EDS mapping results confirm that the IZO and AZO layers can also serve as effective barriers (Figs. S17 and S18) to prevent interlayer ion diffusion. We also tested alternative low-cost metal components in the composite electrode by using low-cost metals such as aluminum (Al) and nickel (Ni), which are also favorable for low-cost PSCs. As the energy level diagram (Fig. S19) shows, the insertion of ITO between the metal and HTL helps to improve the energy level alignment for the PSC. We fabricated PSCs with ITO + Al (ITA) and ITO + Ni (ITN) composite electrodes and tested their long-term stability. The devices with ITA and ITN composite electrodes maintained 95.1 and 91.2% of their initial PCEs after 1000 h, while the corresponding reference devices with only Al and Ni electrodes decayed to almost no efficiency and 56.4% of their initial PCE, respectively, which demonstrates the generality of the composite electrode strategy (Figs. S20 and S21).

## Conclusions

In conclusion, we proposed a composite electrode strategy combined transparent conducting oxide with low-cost metal to improve the long-term stabilities and photovoltaic performances of PSCs. We achieved a champion PCE of 23.7% with a *n-i-p* structure device and maintained more than 90% of the initial PCE after continuous light soaking for 1000 h. Moreover, the composite electrode enables devices with excellent electrical stability under both forward and reverse bias. Systematic characterizations revealed that ITO in the IC electrode functions well as a barrier to effectively suppress the interlayer ion diffusions and defect formation, which bring about all-around efficiency and stability improvement. Furthermore, the composite electrode strategy can be extended to combinations of other TCOs and low-cost metals. The composite electrode strategy opens a new venue to achieve high-performance stable and low-cost perovskite optoelectronics in the future.

### Supplementary Information

Below is the link to the electronic supplementary material.

Supplementary file1 (PDF 1643 KB)

## References

[1] Green MA, Ho-Baillie A, Snaith HJ (2014). The emergence of perovskite solar cells. Nat. Photon..

[2] Correa-Baena JP, Saliba M, Buonassisi T, Grätzel M, Abate A (2017). Promises and challenges of perovskite solar cells. Science.

[3] Yoo JJ, Seo G, Chua MR, Park TG, Lu Y (2021). Efficient perovskite solar cells via improved carrier management. Nature.

[4] Karlsson M, Yi Z, Reichert S, Luo X, Lin W (2021). Mixed halide perovskites for spectrally stable and high-efficiency blue light-emitting diodes. Nat. Commun..

[5] Rong Y, Hu Y, Mei A, Tan H, Saidaminov MI (2018). Challenges for commercializing perovskite solar cells. Science.

[6] Zhao WJ, Xu J, He K, Cai Y, Han Y (2021). A special additive enables all cations and anions passivation for stable perovskite solar cells with efficiency over 23%. Nano-Micro Lett..

[7] Lan D, Green MA (2022). Combatting temperature and reverse-bias challenges facing perovskite solar cells. Joule.

[8] Ni Z, Jiao H, Fei C, Gu H, Xu S (2021). Evolution of defects during the degradation of metal halide perovskite solar cells under reverse bias and illumination. Nat. Energy.

[9] Khenkin MV, Katz EA, Abate A, Bardizza G, Berry JJ (2020). Consensus statement for stability assessment and reporting for perovskite photovoltaics based on ISOS procedures. Nat. Energy.

[10] Bowring R, Bertoluzzi L, O'Regan BC, McGehee MD (2018). Reverse bias behavior of halide perovskite solar cells. Adv. Energy Mater..

[11] Zhang X, Shen T, Guo D, Tang LM, Yang K (2020). Reviewing and understanding the stability mechanism of halide perovskite solar cells. InfoMat.

[12] Boyd C, Cheacharoen R, Bush KA, Prasanna R, Leijtens T (2018). Barrier design to prevent metal-induced degradation and improve thermal stability in perovskite solar cells. ACS Energy Lett..

[13] Kato Y, Ono LK, Lee MV, Wang S, Raga SR (2015). Silver iodide formation in methyl ammonium lead iodide perovskite solar cells with silver top electrodes. Adv. Mater. Interfaces.

[14] Tress W, Yavari M, Domanski K, Yadav P, Niesen B (2018). Interpretation and evolution of open-circuit voltage, recombination, ideality factor and subgap defect states during reversible light-soaking and irreversible degradation of perovskite solar cells. Energy Environ. Mater..

[15] Xie S, Feng A, Wang L, Li N, Cheng X (2022). Bulk defect suppression of micrometer-thick perovskite single crystals enables stable photovoltaics. ACS Mater. Lett..

[16] Fu F, Pisoni S, Jeangros Q, Sastre-Pellicer J, Kawecki M (2019). I_2_ vapor-induced degradation of formamidinium lead iodide based perovskite solar cells under heat-light soaking conditions. Energy Environ. Mater..

[17] Lee H, Lee C (2018). Analysis of ion-diffusion-induced interface degradation in inverted perovskite solar cells via restoration of the Ag electrode. Adv. Energy Mater..

[18] Cheng Y, Liu X, Guan Z, Li M, Zeng Z (2021). Revealing the degradation and self-healing mechanisms in perovskite solar cells by sub-bandgap external quantum efficiency spectroscopy. Adv. Mater..

[19] Li N, Feng A, Guo X, Wu J, Xie S (2021). Engineering the hole extraction interface enables single-crystal MAPbI_3_ perovskite solar cells with efficiency exceeding 22% and superior indoor response. Adv. Energy. Mater.

[20] Lin CT, Ngiam J, Xu B, Chang YH, Du T (2020). Enhancing the operational stability of unencapsulated perovskite solar cells through Cu-Ag bilayer electrode incorporation. J. Mater. Chem. A.

[21] Jeong G, Koo D, Seo J, Jung S, Choi Y (2020). Suppressed interdiffusion and degradation in flexible and transparent metal electrode-based perovskite solar cells with a graphene interlayer. Nano Lett..

[22] Zhao J, Zheng X, Deng Y, Li T, Shao Y (2016). Is Cu a stable electrode material in hybrid perovskite solar cells for a 30-year lifetime?. Energy Environ. Mater..

[23] X. Li, S. Fu, W. Zhang, S. Ke, W. Song et al., Chemical anti-corrosion strategy for stable inverted perovskite solar cells. Sci. Adv. **6**(51), eabd1580 (2020). 10.1126/sciadv.abd1580 10.1126/sciadv.abd1580 PMC7744079 33328236

[24] Trading economics. https://tradingeconomics.com/commodities

[25] Bush KA, Bailie CD, Chen Y, Bowring AR, Wang W (2016). Thermal and environmental stability of semi-transparent perovskite solar cells for tandems enabled by a solution-processed nanoparticle buffer layer and sputtered ITO electrode. Adv. Mater..

[26] You J, Meng L, Song TB, Guo TF, Yang YM (2016). Improved air stability of perovskite solar cells via solution-processed metal oxide transport layers. Nat. Nanotechnol..

[27] Li M, Zhou J, Tan L, Liu Y, Wang S (2022). Brominated PEAI as multi-functional passivator for high-efficiency perovskite solar cell. Energy Environ. Mater..

[28] Zhu S, Yao X, Ren Q, Zheng C, Li S (2018). Transparent electrode for monolithic perovskite/silicon-heterojunction two-terminal tandem solar cells. Nano Energy.

[29] Jiang Q, Zhao Y, Zhang X, Yang X, Chen Y (2019). Surface passivation of perovskite film for efficient solar cells. Nat. Photon..

[30] Zhang C, Liang S, Liu W, Eickemeyer FT, Cai X (2021). Ti_1_-graphene single-atom material for improved energy level alignment in perovskite solar cells. Nat. Energy.

[31] M. Jeong, I.W. Choi, E.M. Go, Y. Cho, M. Kim et al., Stable perovskite solar cells with efficiency exceeding 24.8% and 0.3-V voltage loss. Science **369**, 1615–1620 (2020). 10.1126/science.abb7167 10.1126/science.abb7167 32973026

[32] Li R, Wang P, Chen B, Cui X, Ding Y (2019). NiO_*x*_/spiro hole transport bilayers for stable perovskite solar cells with efficiency exceeding 21%. ACS Energy Lett..

[33] Xie H, Wang Z, Chen Z, Pereyra C, Pols M (2021). Decoupling the effects of defects on efficiency and stability through phosphonates in stable halide perovskite solar cells. Joule.

[34] Cheng Y, Xie C, Liu X, Zhu G, Li HW (2020). High-power bifacial perovskite solar cells with shelf life of over 2000 h. Sci. Bull..

[35] Wu S, Chen R, Zhang S, Babu BH, Yue Y (2019). A chemically inert bismuth interlayer enhances long-term stability of inverted perovskite solar cells. Nat. Commun..

