# Correction to: Double Layer Composite Electrode Strategy for Efficient Perovskite Solar Cells with Excellent Reverse-Bias Stability

**DOI:** 10.1007/s40820-023-01012-w

**Published:** 2023-02-01

**Authors:** Chaofan Jiang, Junjie Zhou, Hang Li, Liguo Tan, Minghao Li, Wolfgang Tress, Liming Ding, Michael Grätzel, Chenyi Yi

**Affiliations:** 1 State Key Laboratory of Power System, Department of Electrical Engineering, Tsinghua University, Beijing, 100084 People’s Republic of China; 2 Institute of Computational Physics (ICP), ZHAW School of Engineering, Wildbachstr. 21, 8400 Winterthur, Switzerland; 3 Center for Excellence in Nanoscience (CAS), Key Laboratory of Nanosystem and Hierarchical Fabrication (CAS), National Center for Nanoscience and Technology, Beijing, 100190 People’s Republic of China; 4 Laboratory of Photonics and Interfaces, Department of Chemistry and Chemical Engineering, Swiss Federal Institute of Technology Lausanne, 1015 Lausanne, Switzerland

**Correction to: Nano-Micro Lett. (2022) 15:12** 10.1007/s40820-022-00985-4

In the original version of this article, the date of acceptance was displayed incorrectly. The corrected date is shown in this correction.

Accepted: November 15, 2022

