# Supplementary material for: Double Layer Composite Electrode Strategy for Efficient Perovskite Solar Cells with Excellent Reverse-Bias Stability
Source: Nanomicro Lett. 2022 Dec 13;15:12. doi: 10.1007/s40820-022-00985-4 (PMC9747998; doi:10.1007/s40820-022-00985-4)
Supplement: Supplementary file 1 — Supplementary file1 (PDF 1643 KB) [file 40820_2022_985_MOESM1_ESM.pdf]

Supporting Information for

Double Layer Composite Electrode Strategy for Efficient Perovskite Solar Cells with Excellent Reverse-Bias Stability

Chaofan Jiang[1], Junjie Zhou[1], Hang Li[1], Liguo Tan[1], Minghao Li[1], Wolfgang Tress[2], Liming Ding[3], Michael Grätzel[4], Chenyi Yi[1, *]

[1]State Key Laboratory of Power System, Department of Electrical Engineering, Tsinghua University, Beijing, 100084, P. R. China

[2]Institute of Computational Physics (ICP) ZHAW School of Engineering Wildbachstr. 21, Winterthur 8400, Switzerland

[3]Center for Excellence in Nanoscience (CAS), Key Laboratory of Nanosystem and Hierarchical Fabrication (CAS), National Center for Nanoscience and Technology, Beijing, 100190, P. R. China

[4]Laboratory of Photonics and Interfaces, Department of Chemistry and Chemical Engineering, Swiss Federal Institute of Technology Lausanne, Lausanne CH-1015, Switzerland

*Corresponding author. Email: yicy@mail.tsinghua.edu.cn (Chenyi Yi)

S1 Device Fabrication

The cleaned ITO glass substrates were treated with the UV-ozone before spin-coating SnO_2 solution (4000 rpm for 30 s). Afterwards, the substrates were annealed at 150 °C for 30 min in ambient air. Another UV-ozone was done before depositing perovskite films. The perovskite films were fabricated by typical two-step method according to our former report [S1]. We adopted the typical solution two-step method. The PbI_2 solution (1.5 M) was dissolved in mixed solvent (DMF/DMSO=9/1) and spun onto the SnO_2 films at 1500 rpm for 30 s and annealed at 70 °C for 1 min. Then the FAI/MAI/MACl (90mg/ 6.39mg/ 9mg) were dissolved in 1ml IPA and spun onto the PbI_2 films at 2000 rpm for 30 s. The perovskite thin films were annealed at 150 °C for 15 min in ambient air (30~40% RH). After cooling the perovskite films, devices were transferred into a N_2 glovebox. For perovskite surface treatment, PEAI solution in IPA was spin-coated onto the perovskite films at 4000 rpm for 20 s. Subsequently, a thin layer of hole transporting film was deposited by spin coating spiro-OMeTAD solution composed of 80 mg spiro-OMeTAD, 30 μL t-BP and 18 μL LiTFSI solution. Then, 10 nm MoO_x was thermally evaporated as the buffer layer. For the composite electrode, indium tin oxide (ITO) was deposited onto MoO_x by magnetron radio frequency (RF) sputtering with a cylindrical ITO target (10% SnO_2 and 90% In_2O_3) in Ar. We used a 4 inches target, 9 cm sample-to-target distance. The sputtering power was 100 W and the sputtering pressure was 1.5×10^{-3} mbar. Finally, 100 nm of low-cost metal (Cu/Al/Ni) was thermally evaporated onto TCO to form the composite electrode.

S2 Supplementary Figures and Tables

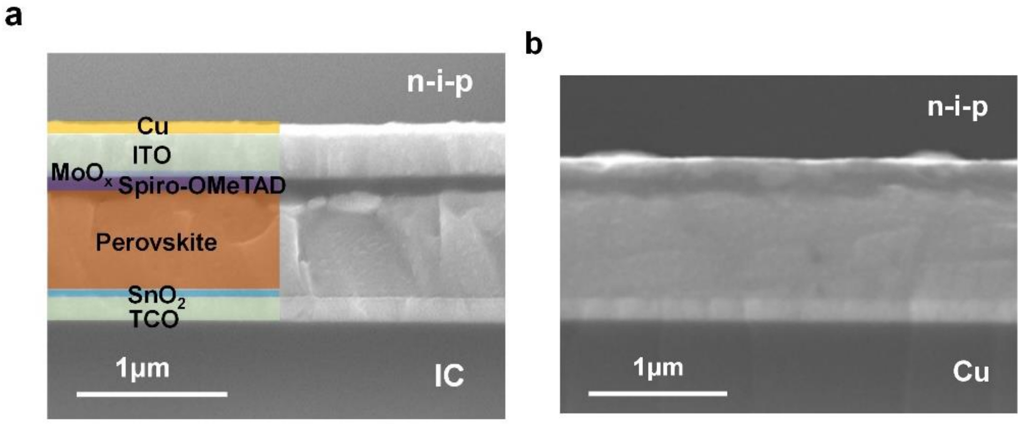

Fig. S1 (**a**) The cross-sectional SEM for IC-PSC. (**b**) The cross-sectional SEM for reference PSC

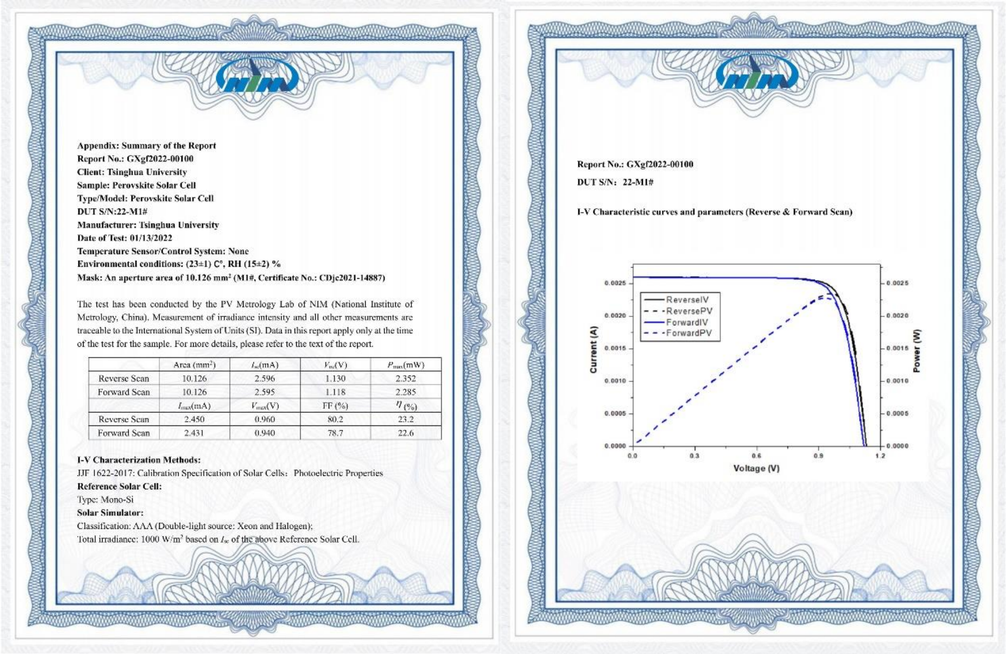

Appendix: Summary of the Report
Report No.: GXgf2022-00100
Client: Tsinghua University
Sample: Perovskite Solar Cell
Type/Model: Perovskite Solar Cell
DUT S/N:22-M1#
Manufacturer: Tsinghua University
Date of Test: 01/13/2022
Temperature Sensor/Control System: None
Environmental conditions: (23±1) C°, RH (15±2) %
Mask: An aperture area of 10.126 mm² (M1#, Certificate No.: CDjc2021-14887)

The test has been conducted by the PV Metrology Lab of NIM (National Institute of Metrology, China). Measurement of irradiance intensity and all other measurements are traceable to the International System of Units (SI). Data in this report apply only at the time of the test for the sample. For more details, please refer to the text of the report.

	Area (mm^2)	I_{sc}(mA)	V_{oc}(V)	P_{max}(mW)
Reverse Scan	10.126	2.596	1.130	2.352
Forward Scan	10.126	2.595	1.118	2.285
	I_{max}(mA)	V_{max}(V)	FF (%)	η (%)
Reverse Scan	2.450	0.960	80.2	23.2
Forward Scan	2.431	0.940	78.7	22.6

I-V Characterization Methods:
JJF 1622-2017: Calibration Specification of Solar Cells: Photoelectric Properties
Reference Solar Cell:
Type: Mono-Si
Solar Simulator:
Classification: AAA (Double-light source: Xeon and Halogen);
Total irradiance: 1000 W/m² based on I_{sc} of the above Reference Solar Cell.

Report No.: GXgf2022-00100
DUT S/N: 22-M1#

I-V Characteristic curves and parameters (Reverse & Forward Scan)

Fig. S2 Certification results of IC-PSCs by the National Institute of Metrology, China (NIM, China)

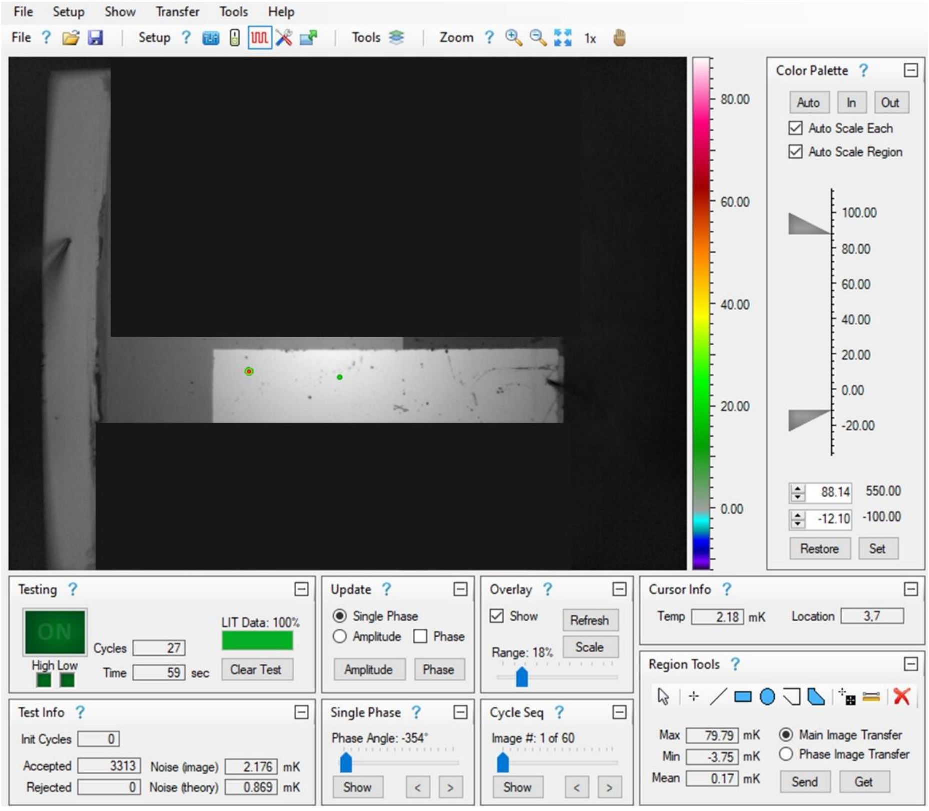

Fig. S3 Thermal microscopy image for reference PSC. Test results (Pulse cycles: 27, Reverse bias voltage: 0.5 V, Leakage current: 1.2 mA, Maximum temperature increase: 79.79 mK)

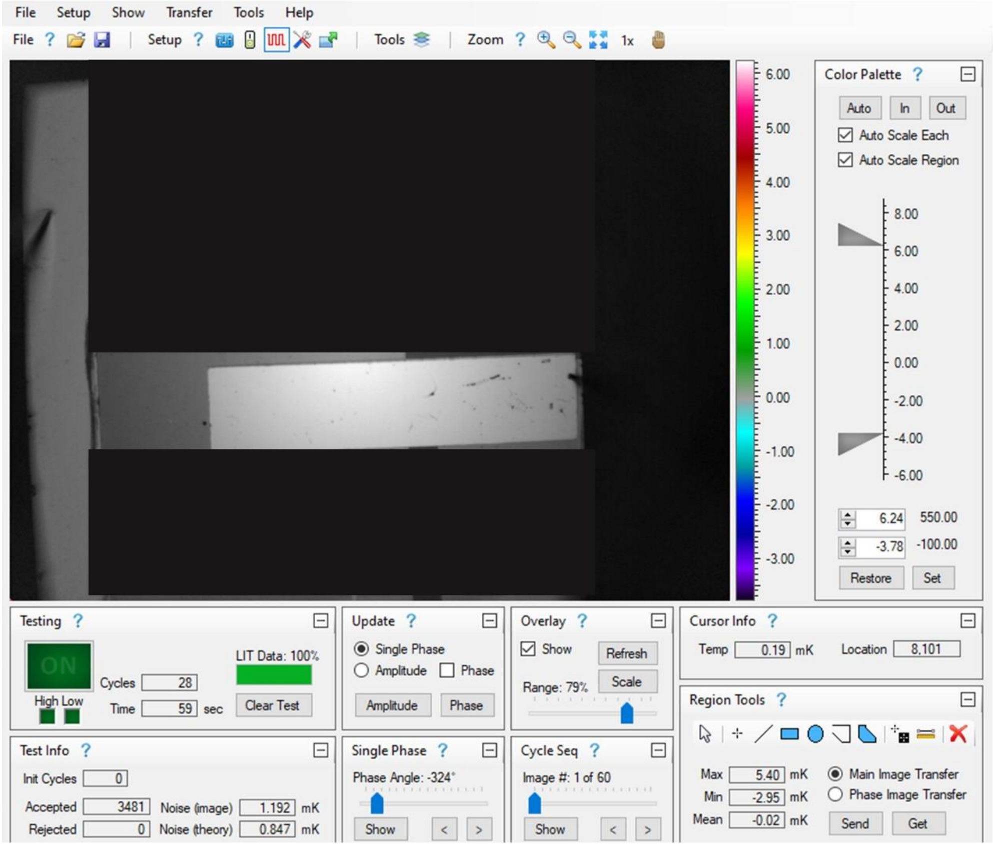

Fig. S4 Thermal microscopy image for IC-PSC. Test results (Pulse cycles: 28, Reverse bias voltage: 1 V, Leakage current: 28 nA, Maximum temperature increase: 5.40 mK)

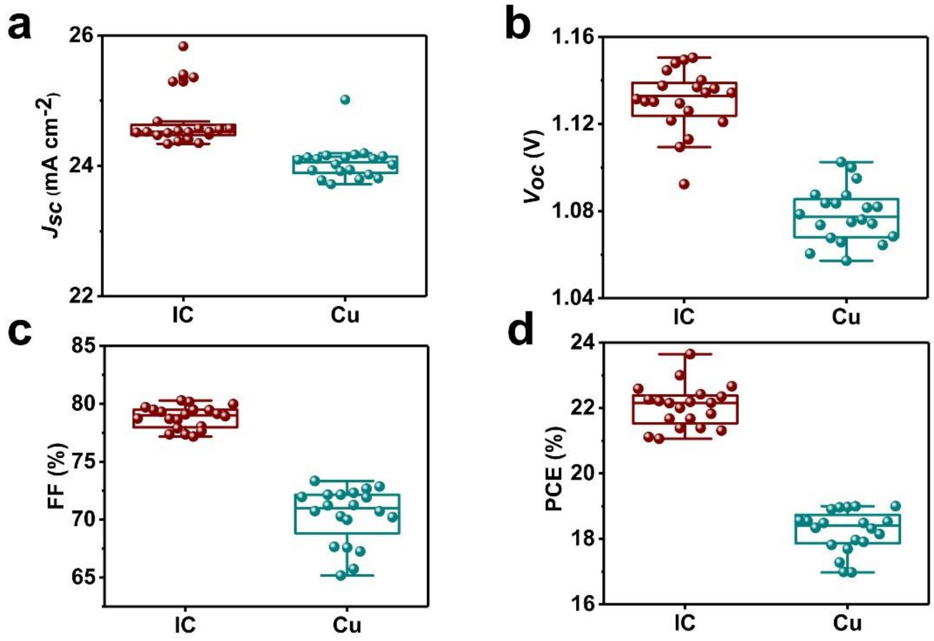

Fig. S5 Device performance metrics statistics (**a**) J_{sc}, (**b**) V_{oc}, (**c**) FF, (**d**) PCE for IC-PSCs and reference PSCs

We fabricated three ITO films with different thicknesses, namely 100, 300 and 500 nm. As shown in Table. 1, the 100 nm ITO showed a relatively low mobility and high sheet resistance; and the 500 nm ITO showed a comparable resistance to 300 nm ITO. Compared with 300 nm ITO, the 500 nm ITO film exhibited a lower mobility and higher carrier concentration, contributing to a stronger absorption of light, which is unfavorable for the light management of PSCs. As a result, best PCE has been achieved with the PSCs based on 300 nm ITO and 100 nm copper as the composite electrode.

Table S1 ITO thickness optimization results

ITO thickness (nm)	Sheet resistivity (Ω/□)	Mobility (cm^2/V s)	Sheet carrier concentration (1/cm^2)	Average PCE
100±10	2753	1.30	1.74E+14	20.83
300±10	93.63	14.71	4.53E+15	22.05
500±10	94.12	10.94	6.06E+15	21.43

We measured the transmittance (T%) for optimized ITO thin film. It is obvious that the film has 76.16% average transmittance from 300 to 900 nm and T% has covered the 80% and approached the 95% at the wavelength of 650 nm.

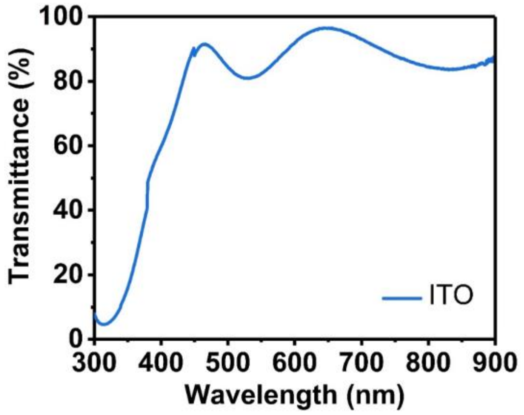

Fig. S6 Transmittance of optimized ITO film

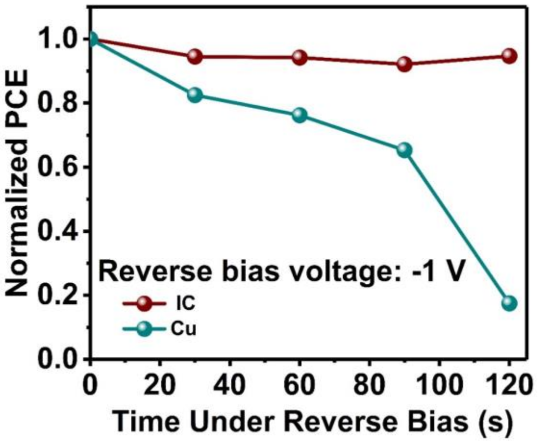

Fig. S7 Reverse-bias stability of the IC-PSC and reference PSC

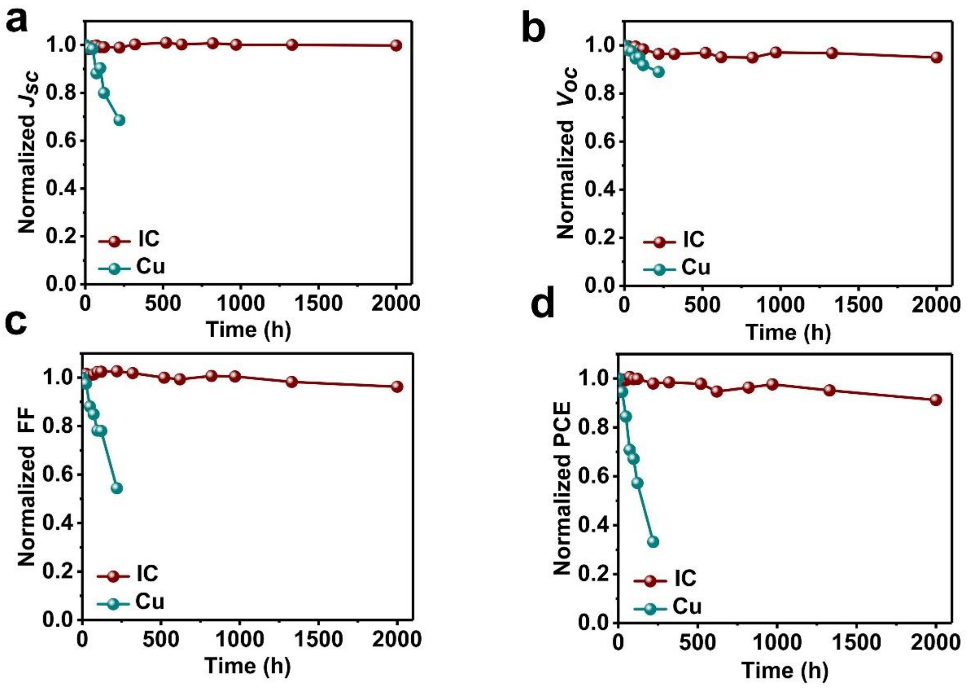

Fig. S8 Photovoltaic performance metrics for devices stored on the shelf. (**a**) normalized J_{sc}, (**b**) normalized V_{oc}, (**c**) normalized FF, (**d**) normalized PCE for IC-PSC and reference PSC

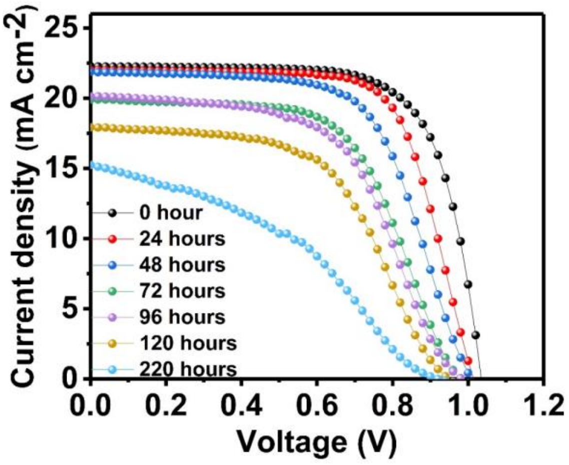

Fig. S9 The *J*-*V* curves for the reference device stored on the shelf (from 0 to 220 hours)

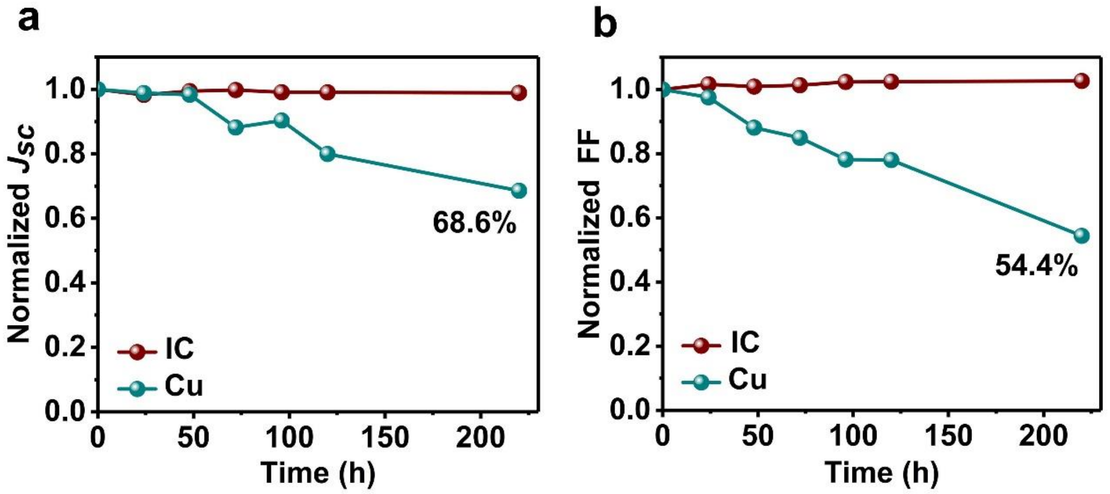

Fig. S10 The key metrics performance changes (**a**) short-circuit current density and (**b**) fill factor of the PSCs in the first 220 h

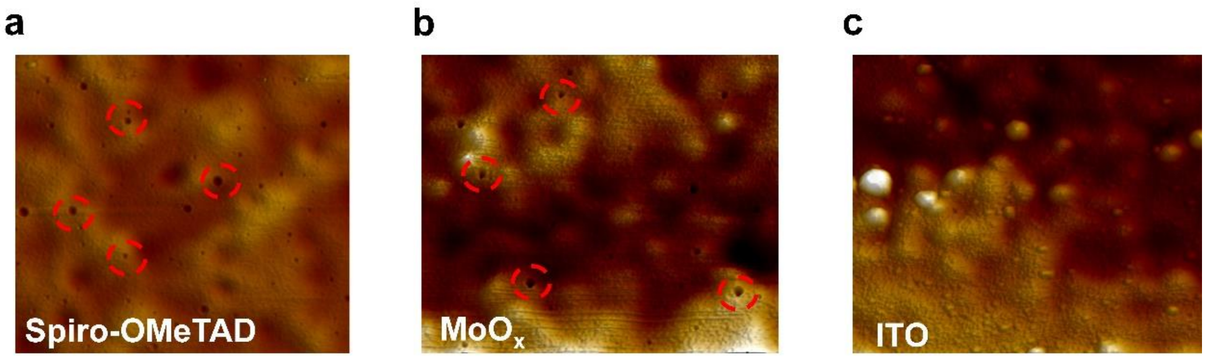

Fig. S11 The 3D visual AFM for the (**a**) Spiro-OMeTAD, (**b**) MoO_x and (**c**) ITO surface morphology

Table S2 The formation enthalpy ($\Delta_f H$) and the free energy ΔE ($E_{M\text{-}M}$–$E_{M\text{-}I}$) of AuI; AgI and CuI [S2]

	$\Delta_f H$	ΔE
AuI	0 kJ mol^{-1}	–49.8 kJ mol^{-1}
AgI	–61.8 kJ mol^{-1}	–71.1 kJ mol^{-1}
CuI	–68.7 kJ mol^{-1}	–88 kJ mol^{-1}

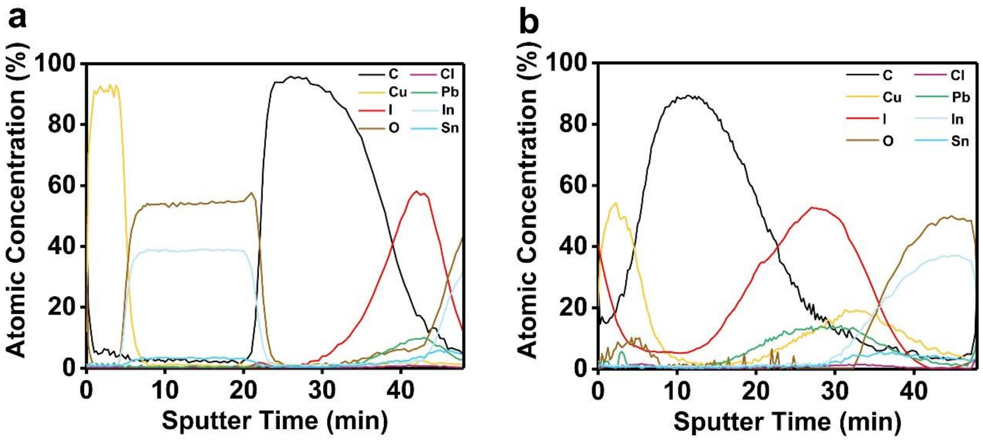

Fig. S12 AES depth element profiling for aged (**a**) IC-PSC and (**b**) reference PSC after 1000 hours MPPT

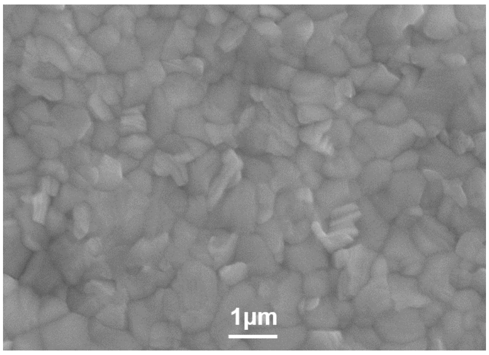

Fig. S13 SEM images of the fresh perovskite film

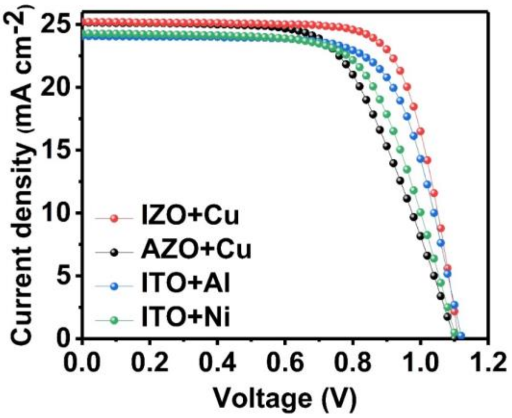

Fig. S14 The PSCs J-V curves based on different composite electrodes

Table S3 Photovoltaic performance of the PSCs based on different composite electrodes

Device	J_{sc} (mA cm^{-2})	V_{oc} (V)	FF (%)	PCE (%)
TCO/SnO_2/Perovskite/SpiroOMeTAD/MoO_x/**IZO+Cu**	25.19	1.11	73.97	20.7
TCO/SnO_2/Perovskite/SpiroOMeTAD/MoO_x/**AZO+Cu**	25.19	1.10	61.85	17.1
TCO/SnO_2/Perovskite/SpiroOMeTAD/MoO_x/**ITO+Al**	24.06	1.12	69.99	18.9
TCO/SnO_2/Perovskite/SpiroOMeTAD/MoO_x/**ITO+Ni**	24.25	1.10	66.20	17.7

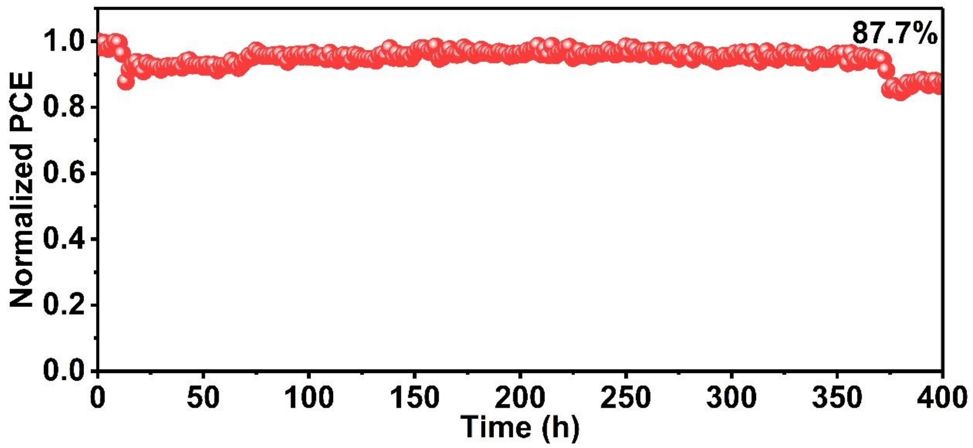

Fig. S15 The MPP tracking performance of the unencapsulated PSCs with IZO+Cu composite electrode under continuous illumination (white LED lamp). The initial PCE was 20.2%

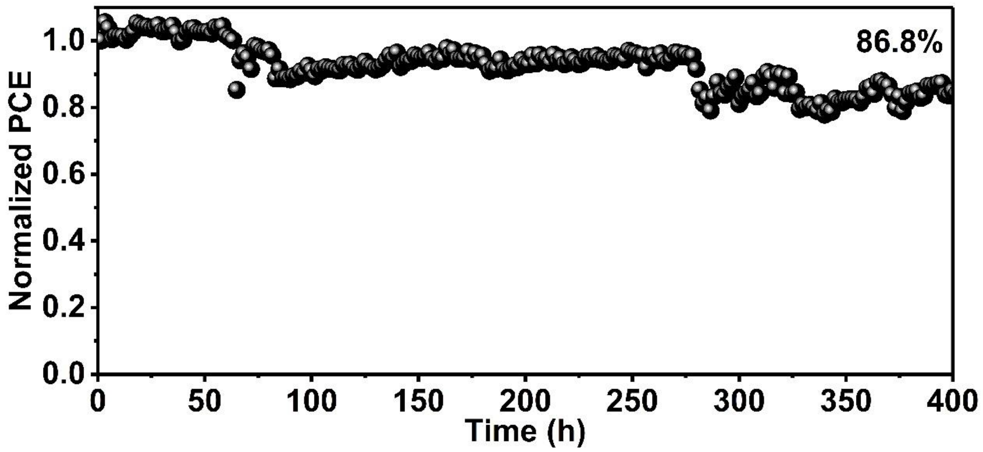

Fig. S16 MPP tracking performance of the unencapsulated PSCs with AZO+Cu composite electrode under continuous illumination (white LED lamp)

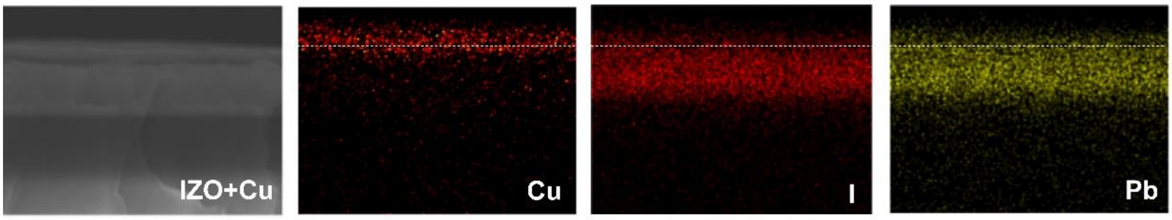

Fig. S17 Cross-section EDX mapping of Cu, I, and Pb elements for PSCs with IZO+Cu composite electrode

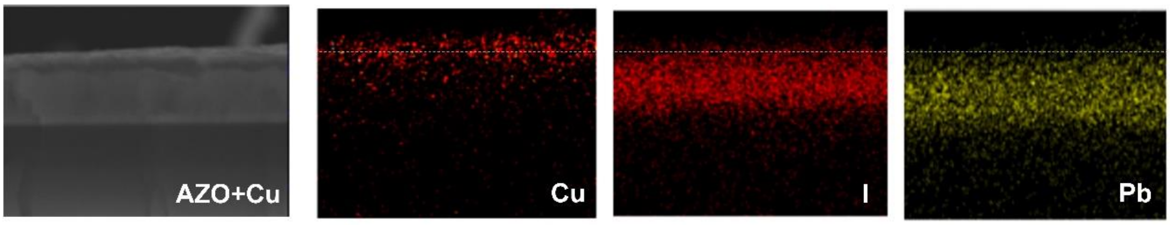

Fig. S18 Cross-section EDX mapping of Cu, I, and Pb elements for PSCs with AZO+Cu composite electrode

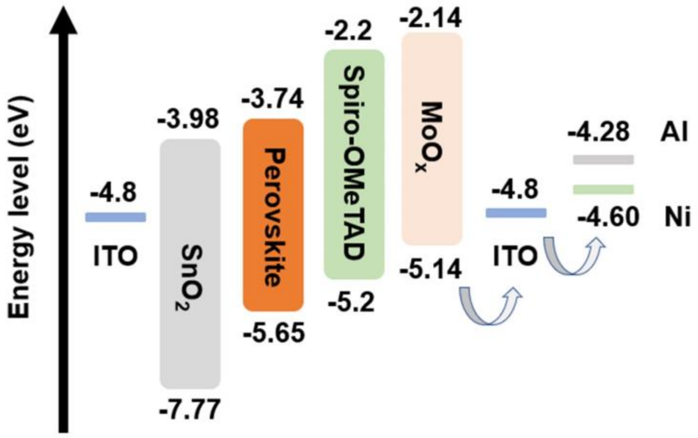

Fig. S19 Energy level matching diagram for PSCs with different metal electrode layer aluminum (Al) and nickel (Ni)

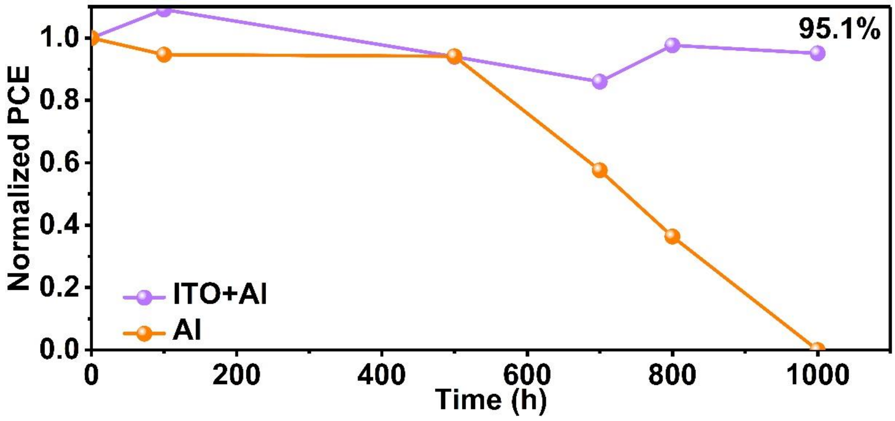

Fig. S20 Device shelf life stability performance for PSCs with ITO+Al composite electrode and reference PSCs

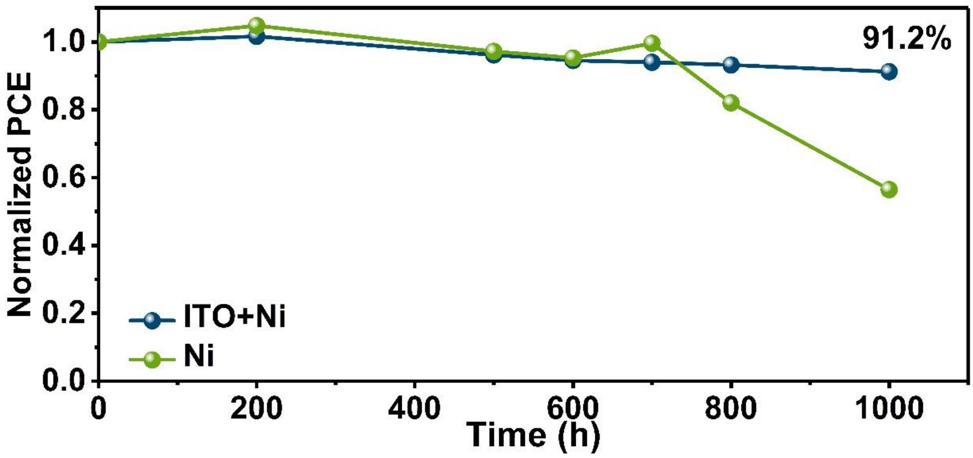

Fig. S21 Device shelf life stability performance for PSCs with ITO+Ni composite electrode and reference PSCs

Supplementary References

[S1] M. Li, J. Zhou, L. Tan, Y. Liu, S. Wang et al. Brominated PEAI as multi-functional passivator for high-efficiency perovskite solar cell. Energy Environ. Mater. (2022). https://doi.org/10.1002/eem2.12360

[S2] S. Wu, R. Chen, S. Zhang, B.H. Babu, Y. Yue et al. A chemically inert bismuth interlayer enhances long-term stability of inverted perovskite solar cells. Nat. Commun. **10**, 1161 (2019). https://doi.org/10.1038/s41467-019-09167-0
